# Measuring and addressing health equity: an assessment of cancer center designation requirements

**DOI:** 10.1007/s10552-023-01680-4

**Published:** 2023-03-20

**Authors:** Jason T. Semprini, Caitlin B. Biddell, Jan M. Eberth, Mary E. Charlton, Sarah H. Nash, Katherine A. Yeager, Donoria Evans, Purnima Madhivanan, Heather M. Brandt, Natoshia M. Askelson, Aaron T. Seaman, Whitney E. Zahnd

**Affiliations:** 1 Department of Health Management and Policy, College of Public Health, University of Iowa, 145 N. Riverside Dr. N277, Iowa City, IA 52240 USA; 2 Department of Health Policy and Management, Gillings School of Public Health, University of North Carolina at Chapel Hill, Chapel Hill, NC USA; 3 Department of Health Management and Policy, Dornsife School of Public Health, Drexel University, Philadelphia, PA USA; 4 Department of Epidemiology, College of Public Health, University of Iowa, Iowa City, IA USA; 5 Department of Community and Behavioral Health, College of Public Health, University of Iowa, Iowa City, IA USA; 6 Nell Hodgson Woodruff School of Nursing, Emory University, Atlanta, GA USA; 7 National Partnerships and Innovations, American Cancer Society, Decatur, GA USA; 8 Department of Health Promotion Sciences, Mel & Enid Zuckerman College of Public Health, University of Arizona. Tucson, Tuscon, AZ USA; 9 St. Jude Children’s Research Hospital and St. Jude Comprehensive Cancer Center, Memphis, TN USA; 10 Department of Internal Medicine, Carver College of Medicine, University of Iowa, Iowa City, IA USA

**Keywords:** Equity, Cancer centers, Quality, Diversity, Treatment

## Abstract

**Purpose:**

By requiring specific measures, cancer endorsements (e.g., accreditations, designations, certifications) promote high-quality cancer care. While 'quality' is the defining feature, less is known about how these endorsements consider equity. Given the inequities in access to high-quality cancer care, we assessed the extent to which equity structures, processes, and outcomes were required for cancer center endorsements.

**Methods:**

We performed a content analysis of medical oncology, radiation oncology, surgical oncology, and research hospital endorsements from the American Society of Clinical Oncology (ASCO), American Society of Radiation Oncology (ASTRO), American College of Surgeons Commission on Cancer (CoC), and the National Cancer Institute (NCI), respectively. We analyzed requirements for equity-focused content and compared how each endorsing body included equity as a requirement along three axes: structures, processes, and outcomes.

**Results:**

ASCO guidelines centered on processes assessing financial, health literacy, and psychosocial barriers to care. ASTRO guidelines related to language needs and processes to address financial barriers. CoC equity-related guidelines focused on processes addressing financial and psychosocial concerns of survivors, and hospital-identified barriers to care. NCI guidelines considered equity related to cancer disparities research, inclusion of diverse groups in outreach and clinical trials, and diversification of investigators. None of the guidelines explicitly required measures of equitable care delivery or outcomes beyond clinical trial enrollment.

**Conclusion:**

Overall, equity requirements were limited. Leveraging the influence and infrastructure of cancer quality endorsements could enhance progress toward achieving cancer care equity. We recommend that endorsing organizations 1) require cancer centers to implement processes for measuring and tracking health equity outcomes and 2) engage diverse community stakeholders to develop strategies for addressing discrimination.

## Introduction

Inequities across the cancer care continuum remain a major challenge in the United States [1]. Despite recent healthcare reforms which have increased access to insurance, low-income adults with Medicaid coverage face barriers to accessing cancer care at high-quality centers [2]. Moreover, even after accessing a cancer center for treatment, uninsured or publicly insured cancer patients are less likely to receive recommended cancer treatment than privately insured counterparts [3]. In addition to socioeconomic and insurance status, many people who represent minoritized racial/ethnic groups and other medically underserved populations experience a disproportionate burden of poor cancer outcomes [4]. For instance, despite lower cancer incidence than the general population, Non-Hispanic Black and American Indian/Alaska Native people have the highest all-cancer mortality rates [5]. Disparities in access to high-quality, guideline concordant care likely contribute to racial cancer mortality disparities [6–8]. Similarly, many individuals experience structural barriers to quality cancer care that exacerbate inequities, including those living in poverty or in rural areas; as a result of this and other contributing factors, cancer mortality rates are higher in rural populations, and among those of lower socioeconomic status [9–11]. To address these challenges, health systems have begun prioritizing initiatives to achieve health equity [12, 13].

The U.S. Department of Health and Human Services (HHS) have defined health equity as “the attainment of the highest level of health for all people” [14]. The HHS further states that, “Achieving health equity requires valuing everyone equally with focused and ongoing societal efforts to address avoidable inequalities, historical and contemporary injustices, and social determinants of health—and to eliminate disparities in health and health care.” [14]. Braveman and colleagues, in a Robert Wood Johnson Foundation report, additionally stressed that health equity is a process which must be actionable and measurable to be accountable [15]. One strategy to hold health systems accountable for implementing health priorities is to leverage public regulations or private endorsements (i.e., accreditations, certifications, and designations). At a system level, cancer center accreditations and designations offer an opportunity to enact standards for integrating health equity into clinical care and research practice**.** These endorsing organizations have the influence and infrastructure needed to require cancer centers to measure, track, and report outcomes to ensure, among other quality-measures, progress toward equity. Failing to leverage the influence of cancer center endorsements to advance equity would be a missed opportunity.

Araujo and colleagues’ (2020) systematic review of the impact of hospital accreditation on healthcare quality found that accreditation may have a positive impact on efficiency, safety, effectiveness, timeliness, and patient-centeredness [16]. Yet, to our knowledge, no studies have investigated the inclusion of equity in cancer endorsement requirements. The objective of this study was to identify the extent to and ways in which health equity is incorporated into the standards, guidelines, and recommendations for cancer center accreditation and designations.

## Methods

We performed a qualitative, directed content analysis of cancer-relevant hospital accreditations and designations [17, 18]. We selected four accreditations/designations that span medical oncology, radiation oncology, surgical oncology, and cancer research: American Society of Clinical Oncology (ASCO) Quality Oncology Practice Initiative (QOPI), American Society of Radiation Oncology (ASTRO) Accreditation Program for Excellence (APEx) standard, American College of Surgeons Commission on Cancer (CoC) accreditation, and National Cancer Institute (NCI) Cancer Center designation [19–22]. While more accrediting/designating bodies exist, we chose these four as they are broadly nationally-representative, and address both the clinical and research missions of cancer programs.

Our data, which we accessed and analyzed between February-June 2022, comprised the standards that each designation/accreditation requires its members to achieve and maintain. Analysis for each accreditation/designation’s content related to health equity was conducted by at least two team members. The research team deductively developed an initial codebook of explicit health equity content, which was based upon the Alcaraz framework for understanding and addressing social determinants to advance cancer and health equity [23]. This codebook included the following terms: equity, disparities, race, barriers, population, vulnerable, rural, disability, sexual orientation, minority, poverty. Using the initial codebook, the smaller teams coded each organization’s standards.

While reviewing each set of standards for the predesignated codes, we also allowed additional terms and concepts related to health equity (e.g., socioeconomic status) to emerge inductively. Furthermore, we identified and coded excerpts of the endorsements that could implicitly be related to health equity. As the Agency for Healthcare Research and Quality (AHRQ) considers equity to be a domain of healthcare quality, we then categorized each equity term or concept under three axes from the Donnabedian model–a seminal healthcare quality model: structures, processes, and outcomes [24, 25]. Finally, through document review and group discussion, we identified opportunities for enhancing equity requirements in the endorsement process. At each step, we convened as a full team to report back, revise the codebook, and resolve discrepancies.

## Results

**Table 1:** Explicit and Implicit Inclusion of Health Equity Content within Cancer Designation Requirements

	Types of Inclusion	Example	ASCO	ASTRO	COC	NCI
Explicit Terms						
Equity						
Disparities	Research	NCI:” briefly describe how the cancer research relevant to the catchment area is addressed. This may include, for example, a discussion of research focused on cancer health disparities”				P
Race	Research	NCI: “Are differences addressed, if applicable, in the intervention effect due to sex/gender and race/ethnicity?”				P
Rural	Research, plan development for improvement, clinical trial enrollment, criteria exception due to rural location	NCI:” briefly describe how the cancer research relevant to the catchment area is addressed. This may include, for example, a discussion of research focused on cancer health disparities” (e.g.……rural)”	S		P, O	P
Vulnerable						
Under-represented	Community engagement through community advisory boards/partnerships; funding of personnel (e.g., patient navigators), clinical trial enrollment	NCI: “Centers are expected to engage communities within their catchment area to decrease their cancer burden, particularly among minority and underrepresented populations. To facilitate these activities, Centers establish community advisory board(s) and partnerships with other healthcare delivery systems and state and community agencies and coalitions for dissemination of evidence-based findings.”				P, S
Disability	Assessment of functional status, processes to identify and communicate with patients with communication barriers	ASCO:”Assess, document, and address psychosocial needs (i.e., mental illness, cognitive/intellectual capabilities; cultural status; financial wellbeing)”	P	P		
Sexual Orientation	Research focus	NCI:” briefly describe how the cancer research relevant to the catchment area is addressed. This may include, for example, a discussion of research focused on cancer health disparities” (e.g.……sexual and gender minorities)”				P
Minority/Minoritized/Marginalized	Research focus	NCI:”briefly describe how the cancer research relevant to the catchment area is addressed. This may include, for example, a discussion of research focused on cancer health disparities” (e.g.……racial/ethnic minorities)”				P
Income/Poverty/SES	Research focus, development / implementation of survivorship program in part addressing financial issues, initial psychosocial assessment, including SES), education RE: treatment plan that addresses potential financial barriers), provide information and assessment on cost of radiation therapy	COC: “1) Requires development / implementation of survivorship program, which could include services to address financial support of a cancer survivor.”	P	P	P, S	P
Diversity	Diversity of personnel, processes for monitoring and evaluating progress in diversity, inclusion in clinical trial enrollment	NCI: “Each center may also have special opportunities within its catchment area to enhance the diversity of its staff, membership and leadership.”				S, P, O
Population	Catchment area engagement, partnerships, inclusion in education and training, materials aligned with education/reading needs of patient population	ASCO: “All materials must be culturally sensitive and address the various educational and reading levels of the population.”	P		P	S, P, O
Barriers	Processes to identify barriers to treatment adherence, development of survivorship programs, palliative services that identify and address psychosocial needs, processes to identify and address language and other communication barriers	ASCO: “Practitioners should be reviewing any barriers to medication adherence and for any issues identified, practitioners should clearly document referrals to other practitioners (e.g., financial counselor or social worker)”	P	P	P, S, O	
Psychosocial	Policies requiring services that address psychosocial needs	ASCO: “Psychosocial Distress Screening: Requires psychosocial services be available on site or by referral. Requires a policy to monitor distress screening and care (provided/referred).”	P, S			
Immigration Status	Policy exemption for reporting	COC: Registry/Data Follow-Up of Patients: CoC explicitly exempts "residents of foreign countries" from follow-up requirements			P	
Language	Translation services	ASTRO: “The ROP has a process for communication with patients who do not speak English fluently or have other communication barriers.”		P		
Implicit Domains						
Community Engagement	Planning and evaluation processes related to center goals, including community engagement, scope/quality/impact of community outreach and engagement, engagement of community members/organizations,	NCI: “Describe how the Center has communicated community needs to Center members and catalyzed research in all areas of science (basic, clinical, translational, and population sciences). Centers … may illustrate with examples of research projects how outreach to and engagement of communities resulted in high-impact science.”				P, S, O
Cultural Competence	Psychosocial assessment and action including cultural background	ASCO: “Initial psychosocial assessment, with action taken when indicated (*This documentation may include the use of cultural background and socioeconomic status*)”	P			
Patient Education	Processes for patient education around chemotherapy treatment	ASCO: “A learning assessment will be documented including needs, abilities, preferences and readiness to learn. Patient education materials should be appropriate for the patient’s reading level/literacy and patient/caregiver understanding. Ideally, documentation should include patient feedback reflecting understanding and engagement”	P			

Table 1 reports the results of our qualitative content analysis reviewing how cancer accreditations consider equity

The “explicit terms” were selected prior to analyzing the accreditations. The “implicit terms” were identified during the content analysis as a relevant topic for promoting equity. We list the types of inclusion and provide an example directly from the accrediting document. Under each organization, we indicate how equity is considered as a requirement for accreditation: P (process), S (structure), or O (outcome). Shaded cells indicate “Not Present”

*ASCO* American Society of Clinical Oncology, *ASTRO* American Society of Radiation, *COC* Commission on Cancer, *NCI* National Cancer Institute.

### American Society of Clinical Oncology Quality Oncology Practice Initiative

ASCO QOPI (2020) certification is for practices serving hematology-oncology patients on an outpatient basis, and more than 300 programs have been certified globally. The QOPI standards manual is a 54-page document that outlines four “domains of responsibility” creating a safe environment-staffing and general policy; treatment planning, patient consent, and education; ordering, preparing, dispensing, and administering chemotherapy; monitoring after chemotherapy is given, including adherence, toxicity, and complications. Each domain comprises standards and sub-standards, which contain text on the requirements and identified outcomes [19]. ASCO standards were developed initially in 2007 by the ASCO QOPI Measures Workgroup comprising academic and community oncologists and have been refined iteratively based upon clinical guidelines from ASCO, the National Comprehensive Cancer Network, and other organizations [26].

ASCO QOPI guidelines focused on the provision of high-quality treatment. Among our pre-selected search terms, we found that ASCO QOPI guidelines only mentioned rural populations, and only in Section (1.6) which references adapting structural triage/referral requirements in areas with limited available providers. While not explicit in reference to rural populations, ASCO’s Section (1.5) does require processes to document managing patient ability to overcome financial or transportation barriers to treatment. This section also notes that applicants can include materials or resources aimed at addressing financial or transportation barriers in their plan.

While few specific terms or populations were identified within the ASCO endorsement, the requirements aiming to address financial, health literacy, and psychosocial barriers to treatment adherence implicitly relate to equity. The key process requirements were to conduct and document assessments of barriers to care, whereas sections relating to addressing barriers (e.g., connecting patients to external resources) were merely suggestions.

In addition to financial barriers, the ASCO guidelines require process measures to identify and document a plan for addressing intellectual and mental health or psychosocial barriers to care. These psychosocial barriers could, as described in the guidelines, include functional and/or performance status as defined by an individual’s ability to perform daily activities, mental illness, and cognitive or intellectual capabilities. When conducting an initial psychosocial screening assessment, Section (2.7) indicates that applicants may implement structural quality measures by including screening for various forms of mental and emotional wellbeing.

ASCO also suggests considering process measures to assess how cultural status or patient health may impact a patient’s ability to adhere to treatment. Section (1.2.6) discusses incorporating the comprehension of the patient and/or patient’s family when detailing the treatment and disease plan. ASCO also describes providing education materials at appropriate reading levels, and further suggests documenting patient feedback to reflect understanding and patient engagement.

### American Society of Radiation Oncology Accreditation Program for Excellence

ASTRO APEx was designed from a report aiming to develop standards to improve quality and safety in radiation oncology [20]. The 2020 ASTRO APEx accreditation is a 12-page document with 16 standards, among which 11 standards include “Level 1 standards” [27]. ASTRO standards were developed based upon a consensus report entitled Safety is No Accident: A Framework for Quality Radiation Oncology Care which incorporated recommendations from the ASTRO Multidisciplinary Quality Assurance Committee [28]. With an emphasis on patient safety, the accreditation spans domains related to patient education and treatment, staffing, facilities, emergency preparedness, and processes for quality improvement [28] (Table 1).

Our review did not identify any specific equity-related terms or populations within the ASTRO accreditation guidelines. And, while ASTRO requires performance measurement and outcomes reporting related to the patient experience, there was no explicit requirement in this Section (16) related to specific populations or related to advancing equity. ASTRO did, albeit at times implicitly, include three domains related to equity: language accessibility, patient education and self-management, and financial toxicity.

In Section (14.2) on patient consent, ASTRO explicitly requires that the applicant have a process for communicating with patients who do not speak English fluently when educating and discussing informed consent. This same section also includes processes for educating the patient on their role to consent and safely managing their therapy/treatment. Regarding financial toxicity, Section (15.3) requires that the applicant offer information about the cost of treatment and include processes for assessing the potential for financial toxicity related to such costs.

### American College of Surgeons Commission on Cancer

The CoC endorsement program sets standards to improve quality of life across the cancer care continuum through institutional infrastructure, data capture, and accountability. The 2020 Optimal Resources for Cancer Care document is 110 pages, categorized into 9 chapters of standards and sub-standards [21]. Accreditation requirements include domains related to facilities and equipment, personnel and services, patient care, data surveillance, education, research, and quality improvement. CoC accreditations were developed by a group of 100 members representing more than 50 national, professional organizations. Volunteer contributors work as part of the CoC Standards Revision Project workgroups to assist in development and revision of standards [21].

CoC accreditation guidelines were process-related and tended to focus on addressing financial and psychosocial concerns of survivors and hospital-identified barriers to care. With the goal of removing barriers to cancer care, the CoC accreditation includes multiple requirements which could be interpreted as considerations for health equity.

First, CoC requires that palliative care be provided on-site or by a referral, but also that the applicant describe a plan to identify and address financial or psychosocial barriers to palliative care. Similarly, CoC requires that centers to develop at least three survivorship programs, and mention that these programs could involve psychosocial, psychiatric, or financial services support. The CoC also requires availability of psychosocial support services, either as a structural measure by providing in-person services or as a process measure by facilitating a referral. Applicants are also required to screen for psychosocial distress, a process measure which must then be monitored for outcomes throughout the course of treatment.

Second, CoC requires that applicants establish a cancer committee to identify at least one barrier to care and develop/implement a plan to address the identified barrier. Most of the CoC accreditation did not include any explicit equity-related terms or marginalized populations. However, in this Sect. (8.9) the CoC accreditation includes an example of a potential barrier and explicitly mentions specific barriers for rural patients (ex: provider shortages, uninsurance, underinsurance). While not required, this section also describes that conducting a Community-Needs Assessment could be a successful process measure to both identify and address barriers to care most commonly identified in the community.

### National Cancer Institute Cancer Center Designation

NCI-designation guidelines, which focus on building cancer research capacity, are drawn from the most recent P30 Cancer Center Support Grant program announcement (PAR-21–321) [22]. In addition to common grant application sections related to budget, research plans, and human subjects, guidelines focused on the grant program purpose, essential characteristics of the program, research areas, research plans, and consortia. The essential characteristics include physical space, organizational capabilities, transdisciplinary collaboration and coordination, cancer focus, institutional commitment, and center director. The NCI Cancer Centers Program was created as part of the National Cancer Act of 1972. Currently, there are 71 NCI-designated centers with the majority of these being comprehensive cancer centers. These centers are required to re-apply for designation status every 5 years based upon the standards set forth in the P30 Cancer Center Support Grant.

NCI designation guidelines considered equity with regards to implementing process measures to advance cancer disparities research and training efforts, identifying factors associated with cancer burden in the catchment area, diversifying affiliated investigators, and broadening inclusion of diverse groups in community engagement activities and clinical trials. In the training plan section, the NCI designation explicitly mentions “health disparities” as a possible component for the applicant’s research training and education plan.

Within the research plan section, the NCI explicitly defines cancer/health disparities. Although not explicitly required to identify or describe health disparities, the NCI does require cancer centers to implement process measures to identify “factors that characterize and influence the cancer burden in the (applicant’s) catchment area”. Among these factors is the explicit option of describing disparities in cancer risk factors. Additionally, the NCI designation application explicitly mentions other populations and equity-related terms from our codebook. We found explicit mention that NCI applicants describe (within their catchment area) demographic factors (race, ethnicity, sex, gender, age), under-represented populations, socioeconomic status, rurality, sexual and gender minority populations.

Since January 2022, the NCI has specifically required applicants to submit a Plan to Enhance Diversity, explicitly stating a commitment to “ensuring all Americans share equally in the medical advances that result from cancer research…and that disparities in the burden of cancer are reduced or eliminated” [22]. Once again, we identified specific terms and populations explicitly mentioned by the NCI: women, minorities, underrepresented groups. Moreover, within this plan, the NCI requires applicants to describe how they will implement structural measures to establish a pipeline to recruit new cancer researchers from historically Black colleges/universities or other minority-serving institutions and implement programs (processes) to achieve outcome measures promoting the careers of women, minorities, and underrepresented groups. These plans require monitoring and evaluation processes to ensure institutions are on track with their commitment to increasing diversity in cancer research and leadership.

Finally, the NCI explicitly states a goal of enhancing the diversity of clinical trial participants and making special considerations for including women and minorities in clinical research. Processes must also be made for including underrepresented populations, such as rural residents, older adults, and persons with low socioeconomic status, as appropriate within the applicant’s catchment area. In addition to explicitly identifying these populations, the NCI designation includes a structural requirement to conduct community engagement as a means for improving the diversity of clinical trial participation and cancer research efforts more broadly.

## Discussion

Our review was among the first to systematically assess the extent to which cancer endorsements required measuring and addressing health equity [29]. We selected four organizations whose requirements span the cancer care continuum and have national representation. Yet, we found little evidence across the four endorsements of explicit requirements promoting health equity. Neither did we consistently find evidence that the four endorsements required cancer centers to measure, report, and address health inequities. The exception was the NCI designation, which contained multiple components not only mentioning health disparities, but also requiring Centers to create plans and promote initiatives to reduce health disparities (e.g., workforce diversity plans). In contrast, while health equity concepts were implicit within the other three endorsements, there were no explicit requirements to address inequities. We do not doubt that these organizations share a commitment to advancing health equity. However, we argue that leveraging the influence of cancer quality endorsements could serve as an integral tool to achieve equity across the cancer continuum, but only if explicitly required. Anything less is a missed opportunity.

Health equity was implicitly included in most endorsement requirements through patient-level assessments of financial distress, health literacy, and psychosocial barriers to care [30, 31]. No plans, however, required cancer centers to identify and monitor within-system disparities in uptake or quality of care between population subgroups. Without this broader view, cancer centers risk overlooking systemic issues and social determinants of health since their focus solely rests on individual patients. This is particularly evident in underrepresented groups (e.g., non-citizens, LGBTQ populations), who may be more likely to fall through the cracks in fragmented health systems where the provider “hands off” the patient to a plethora of other departments (e.g., patient records, billing, and social services) without ensuring they received the expected follow-up. An unfortunate outcome of neglecting upstream and systemic determinants of health is the widening of existing inequities and resultant disparities over time. Thus, it is critical that cancer center endorsements consider the wider context of an individual’s life in the development, and treatment, of their cancer.

The incorporation of equity in cancer-relevant designation were largely process measures with limited focus on structures and outcomes beyond NCI’s focus on community engagement and diversity of investigators. Process measures, like incorporating assessment and documentation of financial needs of patients or barriers to medication adherence, are necessary, but insufficient to address health equity. To advance cancer/health equity, it is imperative to have structures in place to facilitate processes and outcome measures to assess progress toward health equity. For example, to ensure financial needs of patients are adequately addressed, the incorporation of financial navigators and financial navigation training of staff may be an effective way to ensure the equitable implementation of those important processes [32]. It is also important to ensure that patients who may be in need of such support can access care at NCI-designated or CoC-accredited centers. A recent “secret shopper” study showed that some CoC-accredited centers noted that they did not accept new patients on Medicaid [33]. Regardless of how or if patients are insured it is important to ensure they can access necessary care and that their non-insurance needs are also supported.

Research shows that persons from underrepresented groups, including Black, Indigenous, and people of color (BIPOC), and those living in rural areas, may be less likely to adhere to care plans presented during the course of cancer treatment due to a range of structural barriers to care [34–36]. Therefore, it is no surprise that this consideration was reflected in at least one of the endorsing body requirements (i.e., ASCO). However, it is important that health systems put in place mechanisms to ensure that providers do not alter their treatment planning based upon inappropriate assumptions about patients’ ability to adhere to treatment, thereby increasing inequities in cancer care. Moreover, health systems should leverage their EHR or billing data to find suboptimal patterns of care (e.g., differences between racial/ethnic groups in receipt of guideline-concordant treatment) and use quality improvement initiatives, patient navigation, and patient-reported outcome measurement to address identified gaps.

To advance health equity, we recommend that endorsing organizations require structures and processes to explicitly establish a multi-level focus on equity. No plans specifically identified racism, ableism, or discrimination related to gender identity or sexual orientation as a barrier to screening or preventive care, treatment adherence, quality of life, or survivorship outcomes. Addressing racism and other forms of discrimination requires a broad system-level cultural shift, including acknowledgment and commitment to change by leadership. Endorsing bodies have the opportunity to help catalyze such a shift in tangible ways including, e.g., requirements to 1) leverage EHR and/or claims data to document existing inequities in uptake or quality of care, and 2) build partnerships with patient leaders and community members [37, 38]. By requiring partnerships with, and investments in, community and community-engaged activities, in particular, these endorsements could directly confront barriers to addressing racism in and out of the cancer centers. The NCI designation can serve as a model for other endorsing bodies, which could be adapted to further promote and require community-engaged and community-led research and initiatives. To help ensure such efforts to address discrimination are meaningful and not additionally burdensome, we recommend that endorsing bodies establish internal structures to engage community members throughout the endorsing process. Rather than suggesting, endorsing bodies should explicitly require that cancer centers create similar structures to engage diverse stakeholders in their catchment area.

We are, however, encouraged by the recent launch of the National Committee for Quality Assurance (NCQA) health-equity accreditation for health care settings. The NCQA effort will include Health Equity Accreditation and Health Equity Accreditation Plus, giving health systems an actionable framework to improve and prioritize health equity for those they serve. The NCQA effort is fundamentally based on the premise that high-quality care is equitable care. This effort will help to ensure everyone receives the best possible care regardless of their race, gender, sexual orientation, socioeconomic or cultural characteristics. This new standard will employ intentionality to assess unwarranted differences and identify historical bias in the health care delivery system [39]. In addition, this new standard will ensure the voices of those who identify with groups that have been previously and are currently marginalized and minoritized are included to eliminate differences in care. Lastly, actions to promote equitable care will be a priority. The NCQA has put forth an urgently needed effort to achieve high-quality, equitable health care.

We also recommend that endorsing organizations require cancer centers to establish processes for measuring and tracking health equity outcomes. The Centers for Medicare and Medicaid Services (CMS), for example, has released a framework for health equity which includes standardization of data collection processes that ensures data on race/ethnicity, sexual orientation, gender identity, etc. are captured [40]. CMS has also added health equity requirements for prospective payment system hospitals beginning in 2023 that include tracking health-related social needs, quality improvement efforts related to health equity, and other strategic requirements [41]. Additionally, the Joint Commission recently released new requirements that include six performance measures around disparities that will be implemented in January 2023 [42]. These requirements are specific to different hospital types (e.g., critical access hospitals) that include establishing leadership to address disparities, assessment of patient social needs, stratifying data by sociodemographic data, action plans, and communication with partners on progress to reduce disparities. Application of this principle on a system or hospital level specific to cancer may facilitate the collection and evaluation of data to ensure that patients, regardless of who they are, receive optimal care and achieve equitable health outcomes.

## Limitations

This study is not without limitations. Although the four selected endorsing organizations span a range of research and oncology areas, this should not be considered an exhaustive or comprehensive review of cancer-relevant or broader, non-disease specific accreditations, designations, or standards. We must also acknowledge that, despite finding little evidence of health equity within the endorsement standards/guidelines, our conclusions should not be extended to represent the organizations’ commitment to equity. Further, just because equity-related initiatives are not required does not mean that they are not being implemented across health systems. However, we note that institutions are incentivized to seek accreditations and designations, and that, as such, the endorsing bodies have a concrete opportunity to address inequity. The inclusion of explicit guidelines and standards around the identification and measurement of health inequities and encouragement of strategies to address them (e.g., quality improvement initiatives) in endorsement requirements would be a more effective way to ensure broadscale implementation across cancer centers and minimize any gaps in evidence-based initiatives promoting equity and evince a bold commitment to equity.

## Conclusion

In our review of these four endorsing bodies, we identified numerous missed opportunities for intentional and explicit requirements for advancing health equity in cancer care. The extent to which health equity was considered in these endorsements focused mostly on research diversification and addressing psychosocial and financial barriers to care. We identified opportunities to build upon these guidelines by requiring accredited centers to track equity in quality of care and outcome measurement, and ensuring inclusivity of clinical trial participants, diversity of investigators, and engagement of diverse communities. Leveraging the influence of cancer quality endorsements could serve as an integral tool to achieve equity across the cancer continuum. Given the current state of cancer inequities in the U.S., we believe endorsing bodies can lead change by thoughtful integration of health equity requirements.

## Data Availability

The data for this qualitative content analysis was derived from publicly available, third-party endorsement documents and can be reviewed online [19–22].
